# Real-world experience with the cusp-overlap deployment technique in transcatheter aortic valve replacement: A propensity-matched analysis

**DOI:** 10.3389/fcvm.2022.847568

**Published:** 2022-08-31

**Authors:** Oliver Maier, Kerstin Piayda, Stephan Binnebößel, Nora Berisha, Shazia Afzal, Amin Polzin, Kathrin Klein, Ralf Westenfeld, Patrick Horn, Christian Jung, Malte Kelm, Verena Veulemans, Tobias Zeus

**Affiliations:** ^1^Department of Cardiology, Pulmonology and Vascular Medicine, Medical Faculty, Heinrich Heine University, Düsseldorf, Germany; ^2^CardioVascular Center (CVC) Frankfurt, Frankfurt, Germany; ^3^Cardiovascular Research Institute Düsseldorf (CARID), Medical Faculty, Heinrich Heine University, Düsseldorf, Germany

**Keywords:** aortic stenosis, TAVR, implantation depth, cusp-overlap, permanent pacemaker

## Abstract

**Background:**

The implantation depth (ID) is a critical condition for optimal hemodynamic and clinical outcomes in transcatheter aortic valve replacement (TAVR). The recently recommended cusp-overlap technique (COT) offers optimized fluoroscopic projections facilitating a precise ID. This single-center observational study aimed to investigate short-term clinical performance, safety, and efficacy outcomes in patients undergoing TAVR with self-expandable prostheses and application of COT in a real-world setting.

**Materials and methods:**

From September 2020 to April 2021, a total of 170 patients underwent TAVR with self-expandable devices and the application of COT, while 589 patients were treated from January 2016 to August 2020 with a conventional three-cusp coplanar view approach. The final ID and 30-day outcomes were compared after 1:1 propensity score matching, resulting in 150 patients in both cohorts.

**Results:**

The mean ID was significantly reduced in the COT cohort (−4.2 ± 2.7 vs. −4.9 ± 2.3 mm; *p* = 0.007) with an improvement of ID symmetry of less than 2 mm difference below the annular plane (47.3 vs. 57.3%; *p* = 0.083). The rate of new permanent pacemaker implantation (PPI) following TAVR was effectively reduced (8.0 vs. 16.8%; *p* = 0.028). While the fluoroscopy time decreased (18.4 ± 7.6 vs. 19.8 ± 7.6 min; *p* = 0.023), the dose area product increased in the COT group (4951 ± 3662 vs. 3875 ± 2775 Gy × cm^2^; *p* = 0.005). Patients implanted with COT had a shorter length of in-hospital stay (8.4 ± 4.0 vs. 10.3 ± 6.7 days; *p* = 0.007).

**Conclusion:**

Transcatheter aortic valve replacement using the cusp-overlap deployment technique is associated with an optimized implantation depth, leading to fewer permanent conduction disturbances. However, our in-depth analysis showed for the first time an increase of radiation dose due to extreme angulations of the gantry to obtain the cusp-overlap view.

## Introduction

Transcatheter aortic valve replacement (TAVR) is a fast-growing section in interventional cardiology. In the last decade, TAVR has become a safe and effective alternative to surgical valve replacement (SAVR) to treat symptomatic severe aortic valve stenosis across all surgical risk categories (1–3). Optimized implantation depth (ID) of transcatheter heart valves (THV) is an essential condition for valuable hemodynamic and clinical outcomes. Implantation located too high toward the aorta can result in complicated coronary reaccess, paravalvular leakage, or even valve embolization. In contrast, deep implantation in the left ventricular outflow tract (LVOT) is associated with aortic regurgitation and increased risk of conduction disturbances leading to higher rates of permanent pacemaker implantation (PPI). Despite advanced development of THV design, pre-procedural planning, and progressive implanters’ experience, current PPI rates following TAVR–especially with self-expandable valves–have remained high (4, 5).

In 2020, the manufacturer of the self-expandable THV CoreValve Evolut (Medtronic Inc., Minneapolis, MN, United States) introduced new best practice recommendations for valve deployment, including the cusp-overlap technique (COT). This is a series of procedural steps designed to provide optimized angiographic projections for TAVR with self-expandable devices (6). Application of COT during valve deployment has been shown to result in a reduced risk of interaction with the conduction system below the annular plane and significantly lower PPI rates (7–9). However, there is not sufficient evidence showing correlations to the achievement of an optimized ID as well as potential pitfalls of a more complex implantation process regarding prosthesis repositioning, radiation dose or amount of contrast medium used.

Therefore, this single-center observational study aimed to investigate short-term clinical performance, safety, and efficacy outcomes in patients undergoing transfemoral, self-expandable TAVR with newer-generation CoreValve Evolut THV regarding COT during valve deployment in a real-world setting.

## Materials and methods

### Study population

**FIGURE 1:**
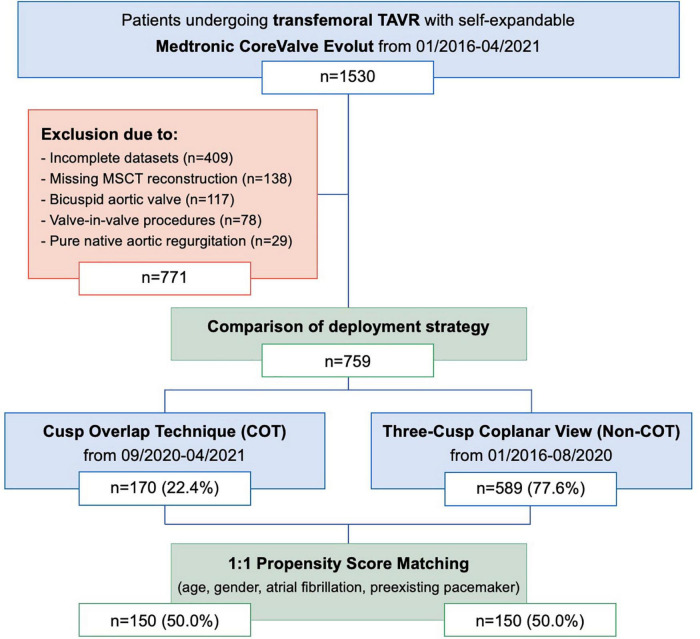
Modified CONSORT Diagram. From January 2016 to April 2021, a total of 1,530 patients underwent TAVR with the Medtronic CoreValve Evolut at the Heart Center Düsseldorf. A total of 759 patients were included in the comparison of the deployment strategy. A total of 1:1 propensity score matches analysis resulted in the final study cohort of 150 COT patients and 150 Non-COT patients. COT, cusp-overlap technique; MSCT, multislice computed tomography; TAVR, transcatheter aortic valve replacement.

From 1530 consecutive patients who underwent transfemoral TAVR with newer-generation self-expandable CoreValve Evolut system (Medtronic Inc., Minneapolis, MN, United States) from January 2016 to April 2021 at the Heart Center Düsseldorf, 759 patients with completed datasets were included in the analysis (Figure 1). Most of the patients excluded from the final analysis due to missing data have lack of documentation in procedural characteristics (pre- and post-dilatation, resheathing, valve dislocation) or post-procedural evaluation of valve function by missing documentation of echocardiography.

**FIGURE 2:**
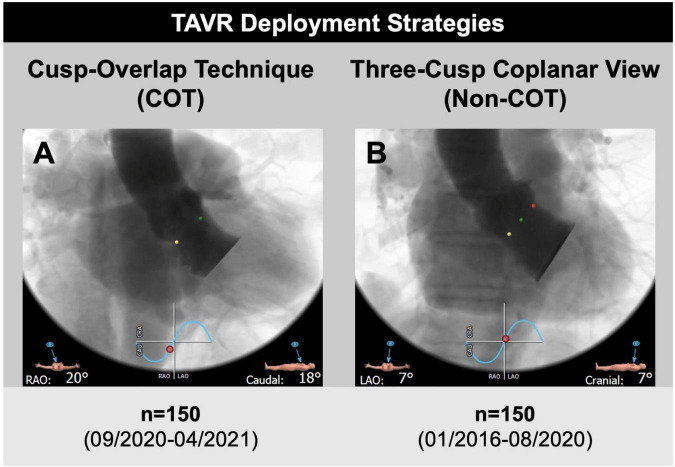
Transcatheter aortic valve replacement (TAVR) deployment strategies. **(A)** A total of 150 patients undergoing transfemoral TAVR with self-expandable Medtronic CoreValve Evolut prostheses and cusp-overlap deployment technique (COT) were compared to **(B)** 150 propensity-matched patients with application of a conventional three-cusp coplanar view only (Non-COT). The cusp-overlap view was generated by overlapping the right coronary cusp (RCC, green dot) and left coronary cusp (LCC, red dot) with isolation of non-coronary cusp (NCC, yellow dot), leading to reduction of parallax in the device and elongation of the left ventricular outflow tract.

The study cohort was further separated into two groups. Patients undergoing TAVR according to Medtronic’s new best practice recommendations of 2020 regarding COT for prosthesis deployment and a target ID of 3 mm were analyzed prospectively from September 2020 to April 2021 (*n* = 170; 22.4%). The control group (Non-COT) treated with former manufacturer’s recommendations without COT but with a conventional three-cusp coplanar view and a target ID of 3–5 mm from January 2016 to August 2020 (*n* = 589; 77.6%) was analyzed retrospectively. To erase potential confounders of the treatment outcome relationship, we performed a 1:1 propensity-score matched analysis resulting in a final study cohort of 150 COT and 150 Non-COT patients (Figure 2). All included patients completed a 30-day follow-up examination to evaluate clinical outcome after TAVR based on Valve Academic Research Consortium-2 (VARC-2) definition (10).

The primary study endpoint was defined as the measurement of ID comparing valve deployment with and without COT during TAVR with self-expandable devices. Target ID is aspired to 3 mm for COT group, compared to target ID of 3–5 mm for Non-COT group, taking into account a measurement uncertainty of 1 mm each.

All participants in this study provided written informed consent. This study was approved by the institutional ethics committee of the Heinrich-Heine University of Düsseldorf and conducted in concordance with the Declaration of Helsinki. The study is registered at clinical trials (NCT01805739).

### 3D image analysis of multislice computed tomography

Multislice computed tomography was routinely performed as native and contrast-enhanced, electrocardiogram gated images. Pre-procedural MSCT data were transferred to a dedicated workstation for 3-dimensional volume-rendered reconstruction (3mensio Structural Heart; Pie Medical Imaging BV, Maastricht, Netherlands). Annular plane projection in the three-cusp view was routinely predicted from MSCT reconstruction and optimal angulation of the cusp-overlap view was generated by overlapping the right coronary cusp (RCC) and the left coronary cusp (LCC) on the MSCT annular plane toward right anterior oblique (RAO) and caudal angulation (6, 11).

### Procedural details

Transcatheter aortic valve replacement procedures were conducted according to current guidelines and under local anesthesia. A total of four experienced operators were involved in this trial. In 2020, Medtronic issued new best practice advice for the CoreValve Evolut system, including cusp-overlap view with isolation of NCC for optimization of the ID during valve deployment. If extreme angulations of more than 30° RAO and/or 30°caudal were suggested for cusp-overlap view, a “near” cusp-overlap view with less extreme angulation was performed. Before final release, a three-cusp view was established to check for complete valve expansion and implantation depth in relation to the left coronary cusp.

The final ID of device implantation was determined angiographically in the three-cusp view after complete clearing of parallax in both COT and Non-COT groups for better visual differentiation of ID below NCC and LCC compared to the cusp overlap view (12). Distance measurements from the interventricular end of the prosthesis to the annular plane were performed afterward using the PACS system workstation (SECTRA IDS7, Linköping, Sweden). As described previously, the arithmetic means of the measured distances from the distal end of the prosthesis to the NCC and the LCC were assessed for final ID (13). Asymmetric valve deployment was defined as a more than 2 mm difference between the NCC and LCC distances. Two independent operators have performed ID measurement. The Pearson correlation coefficient was reported to assess intra- and interobserver reliability for the mean implantation depth measurements from the LCC and the NCC to the prosthesis in 50 randomly chosen cases. Results were interpreted as follows: >0.8, excellent agreement; 0.6 to 0.8, fair to good agreement; 0.4 to 0.6, moderate agreement; and <0.4, no agreement.

### Statistical analysis

Continuous data are described as mean + standard deviation (SD) for normal distribution and comparisons were performed using unpaired Student’s *t*-test or Mann–Whitney *U*-test depending on the variable distribution. Categorical variables are presented as frequencies and percentages and comparisons were made using chi-square or Fisher exact test. All statistical tests were 2-tailed, and a value of *p* < 0.05 was considered statistically significant. To account for the differences in baseline characteristics of COT and Non-COT group, we performed 1:1 propensity score matched analysis using logistic regression and the nearest neighbor method with a caliper of 0.1 because of the observational nature of this study and the comparison of two non-contemporary cohorts with different group sizes. Covariates were chosen according to baseline differences between both cohorts listed in Table 1 (age, gender) as well as previous rhythm and conduction disturbances that could have a disturbing influence on the final analysis regarding new pacemaker dependency following TAVR (atrial fibrillation, preexisting pacemaker). Matching analysis resulted in a final 1:1 matched study cohort of 150 patients in both groups.

**TABLE 1:** Patient clinical and functional characteristics in unmatched cohorts.

	Total (*n* = 759)	COT (*n* = 170)	Non-COT (*n* = 589)	*P*-value
Age, years	81.6 ± 5.6	80.7 ± 6.4	81.9 ± 5.4	*0.018*
Gender, male	385 (50.7)	97 (57.1)	288 (48.9)	*0.061*
BMI, kg/m^2^	26.6 ± 4.7	26.9 ± 5.0	26.6 ± 4.6	0.377
NYHA class III/IV	571 (75.2)	122 (71.8)	449 (76.2)	0.235
CAD	557 (73.4)	113 (66.5)	444 (75.4)	*0.021*
Previous CABG	62 (8.2)	13 (7.7)	49 (8.3)	0.778
Previous valve surgery	5 (0.7)	1 (0.6)	4 (0.7)	0.897
Previous PPI	102 (13.4)	22 (12.9)	80 (13.6)	0.829
Previous LBBB	60 (7.9)	11 (6.5)	49 (8.3)	0.787
Previous RBBB	44 (5.8)	9 (5.3)	35 (5.9)	0.750
Atrial fibrillation	293 (38.6)	50 (29.4)	243 (41.3)	*0.005*
Arterial hypertension	681 (89.7)	147 (86.5)	534 (90.7)	0.113
Diabetes mellitus	176 (23.2)	38 (22.4)	138 (23.4)	0.770
PAD	156 (20.6)	35 (20.6)	121 (20.5)	0.990
Log ES_I, %	22.8 ± 13.7	20.8 ± 12.4	23.4 ± 14.0	*0.020*
STS Score, %	4.9 ± 3.9	4.3 ± 3.3	5.1 ± 4.1	*0.008*
LVEF, %	55.9 ± 12.1	54.9 ± 13.8	56.3 ± 11.6	0.290
CI	2.3 ± 0.5	2.3 ± 0.5	2.2 ± 0.5	0.339
AVA, cm^2^	0.74 ± 0.2	0.75 ± 0.2	0.74 ± 0.2	0.862
dPmean, mmHg	39.0 ± 16.0	41.2 ± 14.4	38.4 ± 16.3	*0.038*
dPmax, mmHg	63.5 ± 23.9	67.0 ± 21.9	62.5 ± 24.4	0.108
Annulus perimeter, mm	76.6 ± 7.3	77.6 ± 7.0	76.4 ± 7.3	*0.051*
Annulus mean diameter, mm	24.3 ± 2.3	24.6 ± 2.2	24.2 ± 2.3	0.100
AVC grading mild	222 (29.3)	45 (26.5)	177 (30.1)	0.366
AVC grading moderate	170 (22.4)	35 (20.6)	135 (22.9)	0.521
AVC grading severe	360 (47.4)	89 (52.4)	271 (46.0)	0.145
LVOT-Calcification	384 (50.6)	94 (55.3)	290 (49.2)	0.164

Values are mean ± SD or *n* (%).

AVA, aortic valve area; AVC, aortic valve calcification; BMI, body mass index; CABG, coronary artery bypass graft; CAD, coronary artery disease; CCS, Canadian cardiovascular society; CI, cardiac index; COPD, chronic obstructive pulmonary disease; CVD, cerebrovascular disease; dPmean/max, mean/maximal transvalvular gradient; LBBB, left bundle branch block; Log ES_I, logistic EuroSCORE I; LVEF, left ventricular ejection fraction; LVOT, left ventricular outflow tract; MSCT, multislice computed tomography; NYHA, New York heart association; PAD, peripheral artery disease; PCI, percutaneous coronary intervention; PPI, permanent pacemaker implantation; RBBB, right bundle branch block; RRT, renal replacement therapy.

All statistical analyses were conducted using SPSS version 23.0 (IBM SPSS Inc., Chicago, IL, United States) and figures created with GraphPad Prism version 8.4 (GraphPad Software, San Diego, CA, United States).

## Results

### Baseline patient characteristics

In the unmatched cohorts (*n* = 759), patients undergoing TAVR with COT were younger (COT 80.7 ± 6.4 years vs. Non-COT 81.9 ± 5.4 years; *p* = 0.018), predominantly male (COT 57.1% vs. Non-COT 48.9%; *p* = 0.061) and had a lower incidence of coronary artery disease (COT 66.5% vs. Non-COT 75.4%; *p* = 0.021) as well as lower surgical risk (STS Score: COT 4.3 ± 3.3% vs. Non-COT 5.1 ± 4.1%; *p* = 0.008) (Table 1). COT patients showed less frequently atrial fibrillation (AF) (COT 29.4% vs. Non-COT 41.3%; *p* = 0.005). A total of 1:1 propensity score matching successfully eliminated any major differences between the two cohorts (Table 2).

**TABLE 2:** Patient clinical and functional characteristics in matched cohorts.

	Total (*n* = 300)	COT (*n* = 150)	Non-COT (*n* = 150)	*P*-value
Age, years	81.3 ± 4.7	81.3 ± 4.7	81.3 ± 4.7	0.999
Gender, male	174 (58.0)	87 (58.0)	87 (58.0)	0.999
BMI, kg/m^2^	26.7 ± 4.7	26.8 ± 5.0	26.5 ± 4.3	0.826
NYHA class III/IV	214 (71.3)	108 (72.0)	106 (70.7)	0.799
CAD	212 (70.7)	104 (69.3)	108 (72.0)	0.612
Previous CABG	30 (10.0)	12 (8.0)	18 (12.0)	0.248
Previous valve surgery	2 (0.7)	1 (0.7)	1 (0.7)	0.999
Previous PPI	24 (8.0)	12 (8.0)	12 (8.0)	0.999
Previous LBBB	19 (6.3)	9 (6.0)	10 (6.7)	0.813
Previous RBBB	15 (5.0)	8 (5.3)	7 (4.7)	0.791
Atrial fibrillation	90 (30.0)	45 (30.0)	45 (30.0)	0.999
Arterial hypertension	267 (89.0)	129 (86.0)	138 (92.0)	0.097
Diabetes mellitus	68 (22.7)	32 (21.3)	36 (24.0)	0.581
PAD	65 (21.7)	31 (20.7)	34 (22.7)	0.674
Log ES_I, %	21.8 ± 11.8	20.9 ± 12.3	22.8 ± 11.4	0.061
STS Score, %	4.5 ± 3.2	4.3 ± 3.4	4.8 ± 3.1	0.062
LVEF, %	55.3 ± 13.1	55.3 ± 13.4	54.7 ± 13.4	0.793
CI	2.3 ± 0.4	2.3 ± 0.5	2.2 ± 0.5	0.643
AVA, cm^2^	0.76 ± 0.2	0.75 ± 0.2	0.77 ± 0.2	0.527
dPmean, mmHg	39.7 ± 14.5	40.9 ± 14.2	38.6 ± 14.7	0.119
dPmax, mmHg	63.5 ± 21.4	65.4 ± 20.0	61.6 ± 22.0	0.087
Annulus perimeter, mm	77.6 ± 7.6	77.5 ± 6.9	77.4 ± 9.2	0.759
Annulus mean diameter, mm	24.6 ± 2.3	24.5 ± 2.2	24.7 ± 2.5	0.524
AVC grading mild	99 (33.0)	47 (31.3)	54 (36.0)	0.392
AVC grading moderate	53 (17.7)	28 (18.7)	30 (20.0)	0.770
AVC grading severe	146 (48.7)	75 (50.0)	66 (44.0)	0.298
LVOT-Calcification	160 (53.3)	80 (53.3)	80 (53.3)	0.999

Values are mean ± SD or *n* (%).

AVA, aortic valve area; AVC, aortic valve calcification; BMI, body mass index; CABG, coronary artery bypass graft; CAD, coronary artery disease; CCS, Canadian cardiovascular society; CI, cardiac index; dPmean/max, mean/maximal transvalvular gradient; LBBB, left bundle branch block; Log ES_I, logistic EuroSCORE I; LVEF, left ventricular ejection fraction; LVOT, left ventricular outflow tract; MSCT, multislice computed tomography; NYHA, New York Heart Association; PAD, peripheral artery disease; PCI, percutaneous coronary intervention; PPI, permanent pacemaker implantation; RBBB, right bundle branch block; RRT, renal replacement therapy.

### Procedural characteristics

After pre-procedural MSCT planning to determine the optimal cusp-overlap projection angle, we achieved a projected cusp-overlap gantry view on CT reconstruction in 121 patients (80.7%) of patients and a near cusp-overlap projection in the remaining 29 cases (19.3%).

**FIGURE 3:**
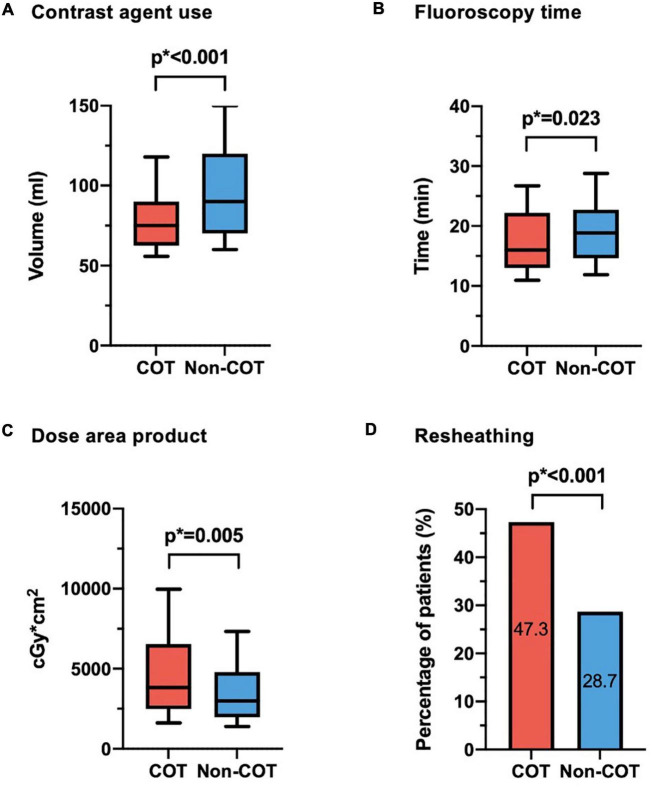
Procedural data. **(A)** Reduction of contrast agent use could be achieved in COT patients (COT 82.8 ± 33.4 ml vs. Non-COT 96.9 ± 33.6 ml; *p* < 0.001). **(B)** While fluoroscopy time was shorter (COT 18.4 ± 7.6 min vs. Non-COT 19.8 ± 7.6 ml; *p* = 0.023), **(C)** radiation dose area product was enhanced in the COT group (COT 4,951 ± 3,662 Gy × cm^2^ vs. Non-COT 3,875 ± 2,775 Gy × cm^2^; *p* = 0.005), probably due to more extreme angulations and **(D)** notably higher rates of device resheathing maneuvers (COT 47.3% vs. Non-COT 28.7%; *p* < 0.001) compared to the Non-COT group. COT, cusp-overlap technique.

Valve size was equally distributed with most of the procedures performed with a 29-mm CoreValve Evolut prosthesis (45.7%). COT patients had less need of contrast agent volume (COT 82.8 ± 33.4 ml vs. Non-COT 96.9 ± 33.6 ml; *p* < 0.001) (Figure 3A) with a reduction in fluoroscopy time (COT 18.4 ± 7.6 min vs. Non-COT 19.8 ± 7.6 ml; *p* = 0.023) (Figure 3B). Patients undergoing TAVR with COT had a significantly higher radiation dose area product compared to the Non-COT group (COT 4951 ± 3662 Gy × cm^2^ vs. Non-COT 3875 ± 2775 Gy × cm^2^; *p* = 0.005) (Figure 3C). COT resulted in notably higher rates of device resheathing compared to the Non-COT group (COT 47.3% vs. Non-COT 28.7%; *p* < 0.001) (Figure 3D). Additional procedural data are shown in Table 3.

**TABLE 3:** Procedural characteristics in matched cohorts.

	Total (*n* = 300)	COT (*n* = 150)	Non-COT (*n* = 150)	*P*-value
Prosthesis size 23 mm	4 (1.3)	2 (1.3)	2 (1.3)	0.999
Prosthesis size 26 mm	73 (24.3)	36 (24.0)	37 (24.7)	0.893
Prosthesis size 29 mm	137 (45.7)	73 (48.7)	64 (42.7)	0.297
Prosthesis size 34 mm	85 (28.3)	38 (25.3)	47 (31.3)	0.249
Contrast agent, ml	89.9 ± 34.2	82.8 ± 33.4	96.9 ± 33.6	< *0.001*
Fluoroscopy time, min	19.1 ± 7.7	18.4 ± 7.6	19.8 ± 7.6	*0.023*
Dose area product, Gy × cm^2^	4413 ± 3288	4951 ± 3662	3875 ± 2775	*0.005*
Pre-dilatation	136 (45.3)	71 (47.3)	65 (43.3)	0.487
Post-dilatation	42 (14.0)	23 (15.3)	19 (12.7)	0.506
Resheathing	114 (38.0)	71 (47.3)	43 (28.7)	< *0.001*
Valve dislocation	4 (0.1)	2 (0.1)	2 (0.1)	0.999
Mean area oversizing,%	7.9 ± 7.4	7.7 ± 6.4	8.1 ± 8.4	0.643
Need for a second transcatheter valve	2 (1.3)	2 (1.3)	0 (0.0)	0.156
Coronary obstruction	0 (0.0)	0 (0.0)	0 (0.0)	0.999
Conversion to surgery	0 (0.0)	0 (0.0)	0 (0.0)	0.999

Values are mean ± SD or *n* (%).

### Procedural and clinical outcome

**FIGURE 4:**
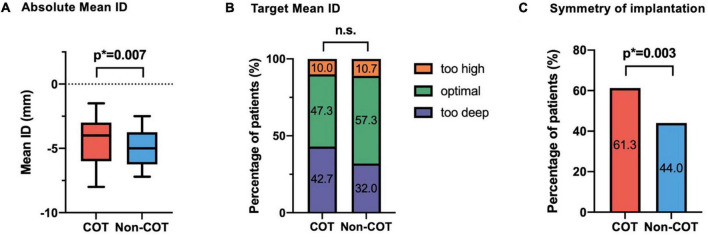
Impact of COT on implantation depth. **(A)** There was a significant reduction of final mean ID in the COT cohort (COT –4.2 ± 2.7 mm vs. Non-COT –4.9 ± 2.3 mm; *p* = 0.007), whereas **(B)** the target ID of 3 mm in the COT group and 3–5 mm in the Non-COT group was reached similarly often. **(C)** Reduction in asymmetric valve deployment with a difference of less than 2 mm between NCC and LCC ID could be achieved more often in the COT group (COT 61.3% vs. Non-COT 44.0%; *p* = 0.003). COT, cusp-overlap technique; ID, implantation depth.

**FIGURE 5:**
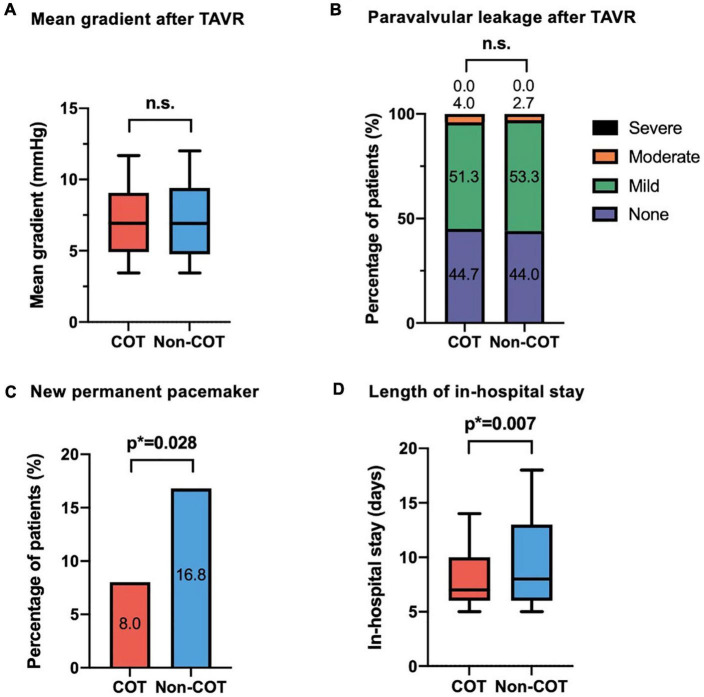
Efficacy outcome of COT. No difference could be observed in **(A)** mean aortic gradient and **(B)** paravalvular leakage following TAVR with different deployment techniques. In the COT group, fewer new permanent pacemaker implantations during 30-day follow-up were needed (COT 8.0% vs. Non-COT 16.8%; *p* = 0.028) **(C)**, and a reduced length of in-hospital stay could be achieved (COT 8.4 ± 4.0 vs. Non-COT 10.3 ± 6.7 days; *p* = 0.007) **(D)**. COT, cusp-overlap technique; TAVR, transcatheter aortic valve replacement.

The final absolute mean ID was significantly reduced in the COT cohort (COT −4.2 ± 2.7 mm vs. Non-COT −4.9 ± 2.3 mm; *p* = 0.007) without a significant difference in the achievement of the target ID of 3 mm in the COT group and 3–5 mm in the Non-COT group (COT 47.3% vs. Non-COT 57.3%; *p* = 0.083) (Figures 4A,B). In COT patients valve deployment was conducted more symmetrically with a difference of less than 2 mm between NCC and LCC ID (COT 61.3% vs. Non-COT 44.0%; *p* = 0.003) (Figure 4C). Both the intra- and the interobserver reliability showed excellent agreement (*r* > 0.8) because we used a standardized measurement technique at our heart center for the analysis of ID after TAVR. Functional improvement was observed in both groups without significant differences concerning mean pressure gradients or paravalvular leakage assessed by TTE during 30-day follow-up (Figures 5A,B).

The rate of new PPI following TAVR was markedly reduced in the COT group (COT 8.0% vs. Non-COT 16.8%; *p* = 0.028) (Figure 5C). The most frequent indication for pacemaker was high degree atrioventricular heart block by far (81 of 90 patients (90%) in the unmatched Non-COT group of 589 patients and 12 of 13 patients (92.3%) in the unmatched COT group of 170 patients). Other indications have been symptomatic bradycardia due to sick sinus syndrome or slowly conducted atrial fibrillation as well as bifascicular block. Therefore, indications for new pacemaker implantation after TAVR did not significantly change over time. Furthermore, the mean time from TAVR to pacemaker implantation has been 2.34 + 0.98 days in the Non-COT group and 2.12 + 0.84 days in the COT group (*p* = 0.289).

There was also an association between COT and lower incidence of new-onset left bundle branch block (LBBB) (COT 12.8% vs. Non-COT 22.9%; *p* = 0.027). Patients implanted with COT had a shorter length of ICU stay (COT 1.6 ± 1.4 days vs. Non-COT 2.3 ± 1.8 days; *p* < 0.001) and a shorter length of total in-hospital stay compared to Non-COT patients (COT 8.4 ± 4.0 vs. Non-COT 10.3 ± 6.7 days; *p* = 0.007) (Figure 5D). All procedures were performed successfully with only one case of 30-day mortality in the Non-COT group (one septic shock during in-hospital stay after TAVR). All 30-day post-procedural outcome parameters according to VARC-2 are shown in Table 4.

**TABLE 4:** A total of 30-day procedural outcome in matched cohorts.

	Total (*n* = 300)	COT (*n* = 150)	Non-COT (*n* = 150)	*P*-value
ID (mean NCC-LCC), mm	−4.6 ± 2.6	−4.2 ± 2.7	−4.9 ± 2.3	*0.007*
Target ID reached	157 (52.3)	71 (47.3)	86 (57.3)	0.083
Symmetric valve deployment	154 (52.7)	92 (61.3)	66 (44.0)	*0.003*
30-day mortality	1 (0.3)	0 (0.0)	1 (0.7)	0.317
Major bleeding	30 (10.0)	11 (12.0)	19 (8.0)	0.124
Major vascular complications	32 (10.7)	20 (13.3)	12 (8.0)	0.135
Stroke	10 (3.3)	7 (4.7)	3 (2.0)	0.198
AKI I-III	39 (13.0)	16 (10.7)	23 (15.3)	0.230
New RRT	3 (1.0)	0 (0.0)	3 (2.0)	0.082
New PPI	34 (12.4) (*n* = 276)	11 (8.0) (*n* = 138)	23 (16.8) (*n* = 138)	*0.028*
New LBBB	50 (14.1) (*n* = 281)	18 (12.8) (*n* = 141)	32 (22.9) (*n* = 140)	*0.027*
New-onset AF	6 (2.9) (*n* = 210)	3 (2.9) (*n* = 105)	3 (2.9) (*n* = 105)	0.999
Need for valve-in-valve procedure	2 (1.3)	2 (1.3)	0 (0.0)	0.156
dPmean, mmHg	7.3 ± 3.3	7.3 ± 3.4	7.3 ± 3.3	0.972
dPmax, mmHg	13.1 ± 6.1	13.2 ± 6.3	13.0 ± 5.8	0.767
PVL > I°	10 (3.3)	6 (4.0)	4 (2.7)	0.520
In-hospital stay, days	9.4 ± 5.6	8.4 ± 4.0	10.3 ± 6.7	*0.007*
ICU stay, days	2.0 ± 1.7	1.6 ± 1.4	2.3 ± 1.8	< *0.001*

Values are mean ± SD or *n* (%).

AF, atrial fibrillation; AKI, acute kidney injury; dPmean/dPmax, mean/maximal transvalvular gradient; ICU, intensive care unit; ID, implantation depth; LBBB, left bundle branch block; LCC, left coronary cusp; NCC, non-coronary cusp; PPI, permanent pacemaker implantation; PVL, paravalvular leakage; RRT, renal replacement therapy.

The outcome results between the unmatched cohorts are in large parts similar to those between the matched cohorts and do not differ in the main results, except for new left bundle branch block following TAVR. This parameter did not reach statistical significance in unmatched cohorts (COT 13.8% vs. Non-COT 16.9%; *p* = 0.364), but finally did after 1:1 propensity score matching (COT 12.8% vs. Non-COT 22.9%; *p* = 0.027). All procedural data and 30-day procedural outcome events of the unmatched study cohorts are shown in Supplementary Tables 1, 2.

## Discussion

Until now, the impact of the recently recommended cusp-overlap technique on the implantation depth and efficacy outcome in a real-world setting using self-expandable devices is still being evaluated with many unknowns. This study demonstrates that application of COT during transfemoral TAVR with self-expandable THV causes.

(1) Optimization of implantation depth by both reducing the mean ID and improving the symmetry of ID between NCC and LCC,

(2) Significant reduction of permanent conduction disturbances with the need for new pacemaker implantation following TAVR,

(3) Consistent quality of hemodynamic outcome regarding valve pressure gradients and paravalvular leakage, and

(4) Increase of radiation dose due to extreme angulations.

After pre-procedural MSCT planning to determine the optimal cusp-overlap projection angle, we achieved a projected cusp-overlap gantry view on CT reconstruction in 81.9% of patients and a near cusp-overlap projection in the remaining cases. Finally, no increase in adverse events during 30-day follow-up could be observed compared to TAVR procedures without the usage of COT and a three-cusp coplanar view during valve deployment instead.

### Improvement of implantation depth caused by cusp-overlap technique

To increase the accuracy of ID with Evolut CoreValve THV, we have performed COT according to the manufacturer’s recommendations since September 2020. In the three-cusp coplanar view, the device is often poorly aligned and there is no clear view of the native annulus relative to the conduction system due to the foreshortening of the LVOT (11). COT view results in an elongation of the LVOT, removement of the delivery catheter parallax, and accentuation of the NCC/RCC commissures in the center of the fluoroscopic view where the conduction system crosses the membranous septum below the atrioventricular node (6, 14). According to the new manufacturer’s advice, Evolut CoreValve’s target ID of 3 mm is recommended instead of a target ID of 3–5 mm as proposed before (15). Due to the wider range of former target ID of 3–5 mm in the Non-COT group (13), the present COT group with a narrow range of 3 mm target ID did not achieve higher proportions of successful target ID, but a significantly higher absolute mean ID was achieved by far. Not only the ID below a single coronary cusp but also the symmetry of valve implantation was improved. This is another benefit of COT during valve deployment because the NCC nadir is better visualized and both the device and the annulus are perpendicularly in-plane without parallax, allowing correct assessment of the true device depth below all three cusps (14).

### Reliability of cusp-overlap technique in safety and clinical outcome

Although balloon-expandable valves exert higher radial forces, rates of new PPI are higher after implantation of self-expandable THV due to differences in the design of the prosthetic frame and technique of implantation. TAVR with supra-annular, newer-generation CoreValve Evolut prostheses have been associated with the need for new PPI in 14.7 to 26.7% of patients within 30 days after the intervention (4, 5).

The proximity of the aortic valve and the cardiac conduction system is one of the reasons for the occurrence of new conduction disturbances following TAVR (16, 17). The close relationship of the left bundle branch to the aortic root explains why LBBB is the most common conduction disturbance following TAVR and why ID greater than the membranous septum length is an independent predictor of PPI (18–20). Interaction of the THV with the surrounding tissue induces either direct injury by radial forces or indirect injury to the conduction tissue by localized edema or hematoma. Consequently, a complete AV block–the main indication for PPI by far in our analysis–may be either the result of total interruption of AV conduction or new-onset LBBB in patients with preexisting RBBB (21, 22).

Our single-center experience using COT for valve deployment showed that only 8% of patients without preexisting pacemaker required new PPI compared to 16.8% in Non-COT group, and the incidence of new-onset LBBB could also be reduced, what is in accordance with former studies (7–9). Because confounders like age and atrial fibrillation were eliminated by propensity score matching and other known procedural predictors like post-dilatation or oversizing did not differ between both groups, the difference in deployment technique seems to be the only reasonable explanation for this improvement in outcome.

A potential limitation of the COT is the implanter’s concern that a shallow THV implantation may lead to a higher rate of valve embolism with upward displacement (“pop-outs”), potentially resulting in increased procedural complexity and patient morbidity (23, 24). In our experience, we did not observe this event in COT group. Both cases with the need for a second valve in the COT group of our study were caused by moderate to severe valvular leakage after implantation of the first TAVR prosthesis due to infolding of the prosthesis and probably incorrect patient-prosthesis sizing.

The reasons for the reduction in length of in-hospital stay in the COT cohort are multifactorial, but one of them is very likely the reduced post-procedural conduction disturbances and therefore the decreased need for a temporary or permanent pacemaker. The clinical impact of new PPI following TAVR has been somehow controversial, but meta-analyses have suggested an increased risk of all-cause mortality at 1 year in patients receiving a new permanent pacemaker and a higher rate of heart failure rehospitalizations (25). Certainly, the requirement for PPI is associated with longer in-hospital stay and increased costs (26), leading to slower periprocedural recovery and increased resource utilization.

### Impact of cusp-overlap technique on procedural performance during transcatheter aortic valve replacement

Similar to our results, there are already former studies examining the effect of COT on conduction disturbances following TAVR with observation of a significant reduction in both LBBB and PPI rate after TAVR (7–9). Mendiz et al. examined primary clinical outcomes without conclusions on procedural data like the achievement of target ID or the impact of COT on procedural aspects like the volume of contrast agent or the radiation dose used (7). Doldi et al. only reported the technical success according to the new 2021 VARC-3 criteria (27) without further procedural details (8). While Pascual et al. performed a propensity score analysis with different measurement techniques of target ID, no relevant information was published about possible procedural pitfalls of this complex implantation technique except for procedural success and fluoroscopic time without any significant difference between COT and Non-COT group (9).

At least in our hands, the price for optimized ID is a higher rate of resheathing due to a more accurate achievement of a target ID of exactly 3 mm than before with a range between 3 and 5 mm. Thus, higher rates of repositioning did not result in less device success or increased mortality of the COT group as described before during multiple resheathing (28). Thus, there is a trend toward increased stroke rates in the COT group compared to Non-COT. Although one could speculate that excessive manipulation in the aortic valve and its adjacent structures could affect stroke rates, no significant relationship has been found in former studies (28, 29). The non-randomized design of our study with different clinical and anatomical factors might have impacted this outcome, and confusion bias cannot be excluded.

Although even the length of fluoroscopy time could be reduced, the dose area product is higher in the COT group, what can be explained by more extreme angulations during cusp-overlap view with an automatic increment of radiation energy that is exposed to the patients’ body surface by the X-ray tube assembly (Figures 3B,C).

A significant reduction of contrast medium was observed in the COT cohort. This is the consequence of the need for less contrast agent usage to visualize the NCC only (2–4 ml) during most valve positioning up to the point of no return. A larger amount of contrast agent is only necessary for the final assessment of valve position before deployment, when, as a second angulation, a three-cusp view is obtained for position control and ID measurement. In contrast, during valve positioning in three-cusp only view, more contrast agent and longer fluoroscopy time were administered to delineate all three cusps.

### Limitations

Several limitations should be considered when interpreting this study. This is a single-center observational study with a limited number of patients. Two non-contemporary groups were compared to assess differences between COT and 3-cusps coplanar view only technique with four different operators performing TAVR procedure with different levels of practical knowledge and experience. There may exist temporal, confounders and selection bias due to high rate of excluded patients with incomplete dataset that was not accounted for in our analysis. Therefore, larger multicenter randomized trials should be conducted to verify the usefulness of COT during THV deployment.

## Conclusion

Transcatheter aortic valve replacement with self-expandable valves using the cusp-overlap deployment technique is associated with an optimized implantation depth, leading to fewer permanent conduction disturbances and a shortened length of in-hospital stay. Any prevention of conduction disturbances can potentially reduce rehospitalizations and late mortality rate, whereas the procedural and functional outcome is not remarkably influenced by COT. Thus, COT should be regularly performed for TAVR with a self-expandable prosthesis to achieve optimized ID, especially as TAVR is expanded to the low-risk population with higher life expectancy.

## Data availability statement

The raw data supporting the conclusions of this article will be made available by the authors, without undue reservation.

## Ethics statement

The studies involving human participants were reviewed and approved by the Institutional Ethics Committee of the Heinrich-Heine University of Düsseldorf (4080). The patients/participants provided their written informed consent to participate in this study.

## Author contributions

OM: conception and design, investigation, analysis and interpretation of data, drafting of the manuscript, project administration, and final approval of the manuscript. KP, SB, NB, SA, AP, RW, PH, and CJ: analysis and interpretation of data, revision of the manuscript for important intellectual content, and final approval of the manuscript. KK: analysis and interpretation of data, data curation, revision of the manuscript for important intellectual content, and final approval of the manuscript. MK: conception and design, validation, drafting of the manuscript, and final approval of the manuscript. VV and TZ: conception and design, investigation, drafting of the manuscript, project administration, and final approval of the manuscript. All authors contributed to the article and approved the submitted version.

## Abbreviations

BEV: balloon-expandable valve
COT: cusp-overlap technique
DLZ: device landing zone
ID: implantation depth
LCC: left coronary cusp
MSCT: multislice computed tomography
NCC: non-coronary cusp
PPI: permanent pacemaker implantation
RCC: right coronary cusp
SAVR: surgical aortic valve replacement
SEV: self-expandable valve
TAVR: transcatheter aortic valve replacement
THV: transcatheter heart valve.
