# Supplementary material for: Real-world experience with the cusp-overlap deployment technique in transcatheter aortic valve replacement: A propensity-matched analysis
Source: Front Cardiovasc Med. 2022 Aug 31;9:847568. doi: 10.3389/fcvm.2022.847568 (PMC9471948; doi:10.3389/fcvm.2022.847568)
Supplement: Supplementary file 1 [file Table_1.pdf]

Supplementary Material

1 Supplementary Data

Supplementary Table 1. Procedural characteristics in unmatched cohorts.

		**Total (n=759)**	**COT (n=170)**	**Non-COT (n=589)**	**p-value**
Prosthesis size	23 mm	9 (1.2)	2 (1.2)	7 (1.2)	0.990
	26 mm	206 (27.1)	42 (24.7)	164 (27.8)	0.418
	29 mm	359 (47.3)	79 (46.5)	280 (47.5)	0.806
	34 mm	184 (24.2)	46 (27.1)	138 (23.4)	0.331
Contrast agent, ml		89.6 ± 42.6	80.7 ± 33.0	92.1 ± 42.8	***0.001**
Fluoroscopy time, min		18.8 ± 9.0	18.1 ± 7.4	18.3 ± 8.8	0.787
Dose area product, Gy*cm^2		4039 ± 3486	5174 ± 4242	3714 ± 3168	***<0.001**
Pre-dilatation		296 (39.0)	72 (42.4)	224 (38.0)	0.309
Post-dilatation		101 (13.3)	27 (15.9)	74 (12.6)	0.262
Resheathing		269 (35.4)	89 (52.4)	180 (30.6)	***<0.001**
Mean area oversizing, %		7.8 ± 7.2	7.6 ± 6.4	8.0 ± 7.9	0.545
Valve dislocation		13 (1.7)	2 (1.2)	11 (1.9)	0.541
Need for a second transcatheter valve		6 (0.8)	2 (1.1)	4 (0.7)	0.519
Coronary obstruction		0 (0.0)	0 (0.0)	0 (0.0)	0.999
Conversion to surgery		0 (0.0)	0 (0.0)	0 (0.0)	0.999

Values are mean ± SD or n (%).

Supplementary Table 2. 30-day procedural outcome in unmatched cohorts.

	**Total (n=759)**	**COT (n=170)**	**Non-COT (n=589)**	**p-value**
ID (mean NCC-LCC), mm	-4.7 ± 2.6	-3.8 ± 2.5	-4.9 ± 2.6	**_*<0.001_**
Target ID reached	424 (55.9)	88 (51.8)	336 (57.0)	0.222
Symmetric valve deployment	399 (52.6)	113 (66.5)	286 (48.6)	**_*<0.001_**
30-day mortality	2 (0.3)	0 (0.0)	2 (0.3)	0.447
Major bleeding	66 (8.7)	15 (8.8)	51 (8.7)	0.946
Major vascular complications	74 (9.7)	22 (12.9)	52 (8.8)	0.111
Stroke	25 (3.3)	7 (4.1)	18 (3.1)	0.495
AKI I-III	99 (13.0)	28 (16.5)	71 (12.1)	0.132
New RRT	13 (1.7)	3 (1.8)	10 (1.7)	0.953
New PPI	105 (16.0)	13 (8.8)	92 (18.1)	**_*0.007_**
	(n=657)	(n=148)	(n=509)	
New LBBB	113 (16.2)	22 (13.8)	91 (16.9)	0.364
	(n=699)	(n=159)	(n=540)	
New-onset AF	23 (4.9)	5 (4.2)	18 (5.2)	0.664
	(n=465)	(n=119)	(n=346)	
dPmean, mmHg	7.1 ± 3.4	7.6 ± 3.4	7.3 ± 3.4	0.311
dPmax, mmHg	13.2 ± 6.2	13.5 ± 6.5	13.1 ± 6.2	0.464
PVL >I°	33 (4.3)	8 (4.7)	25 (4.2)	0.795
In-hospital stay, days	9.7 ± 6.1	8.0 ± 4.9	10.2 ± 6.4	**_*<0.001_**
ICU stay, days	2.0 ± 2.4	1.4 ± 1.0	2.2 ± 2.6	**_*<0.001_**

Values are mean ± SD or n (%).

AF=atrial fibrillation; AKI=acute kidney injury; dPmean/dPmax=mean/maximal transvalvular gradient; ICU=intensive care unit; ID=implantation depth; LBBB=left bundle branch block; LCC=left coronary cusp; NCC=non-coronary cusp; PPI=permanent pacemaker implantation; PVL=paravalvular leakage; RRT=renal replacement therapy.
